# Genome Sequences of Five Clinical Isolates of *Klebsiella pneumoniae*

**DOI:** 10.1128/genomeA.00040-16

**Published:** 2016-03-10

**Authors:** L. Letti Lopez, Brigida Rusconi, Heidi Gildersleeve, Chao Qi, Milena McLaughlin, Marc H. Scheetz, J. Seshu, Mark Eppinger

**Affiliations:** a South Texas Center for Emerging Infectious Diseases, Center for Excellence in Infection Genomics and Department of Biology, The University of Texas at San Antonio, San Antonio, Texas, USA; b Northwestern University Feinberg School of Medicine, Evanston, Illinois, USA; c Department of Pharmacy Practice, Midwestern University Chicago College of Pharmacy, Downers Grove, Illinois, USA; d Department of Pharmacy, Northwestern Memorial Hospital, Chicago, Illinois, USA

## Abstract

*Klebsiella pneumoniae* is a nosocomial pathogen of emerging importance and displays resistance to broad-spectrum antibiotics, such as carbapenems. Here, we report the genome sequences of five clinical *K. pneumoniae* isolates*,* four of which are carbapenem resistant. Carbapenem resistance is conferred by hydrolyzing class A β-lactamases found adjacent to transposases.

## GENOME ANNOUNCEMENT

*Klebsiella pneumoniae* is a Gram-negative, nonmotile, and capsule-forming bacterium that frequently causes nosocomial infections (1, 2). It is a member of a group of bacterial pathogens known as ESKAPE (*Enterococcus faecium, Staphylococcus aureus, K. pneumoniae, Acinetobacter baumannii, Pseudomonas aeruginosa*, and *Enterobacter* species) (3), all of which are multidrug resistant and responsible for a majority of hospital-acquired infections. Strains often exhibit resistance to carbapenems, which have a broad spectrum of activity and are reserved as drugs of last resort, and these strains are becoming increasingly common (4). The therapeutic options for infections caused by these microbes are limited; therefore, there is an urgent need to develop novel treatment strategies to complement the current antimicrobial therapies to battle such broad-range antibiotic-resistant pathogens (5–8).

Whole-genome sequence analysis enables the identification of the molecular basis of antibiotic resistance and facilitates a survey for virulence determinants that can be targeted to reduce the pathogenic effects of the clinical isolates in different models of infection (9–11). To this end, we sequenced the genomes of five clinical isolates of *K. pneumoniae* isolated from patients in a Chicago area hospital.

Total genomic DNA was extracted with the QIAamp DNA minikit, according to the manufacturer’s protocol, and the genomic library was prepared with the TruSeq PCR-free kit with single indexing (12–14). The genomes were sequenced on the Illumina MiSeq platform using a paired-end library with 250-bp read length and assembled into draft genomes with SPAdes 3.5.0 (15). The average G+C content was 57.3%, and the total genome length varied between 5.3 and 5.9 Mbp, which is in accordance with reports for other *K. pneumoniae* genomes and is indicative of the varied mobilome of this species (9). Contigs were annotated using Prokka genome annotation version 1.0.0 (16), predicting on average 5,451 coding sequences, 77 tRNAs, and 7 rRNAs. Annotation revealed the presence of genes associated with a type VI secretion in all genomes (17, 18), while none of the isolates featured virulence factors related to the mucoid phenotype, *rmpA* (19) or *magA* (20), when queried against the UniProt database (21, 22). According to ResFinder (23), all isolates are multidrug resistant and carry resistance loci, e.g., those against aminoglycosides, β-lactams, fluoroquinolones, and sulfonamides, a finding consistent with the strains’ recorded antibiotic resistance phenotypes.

Except for K1, all strains harbor a carbapenemase that was found neighboring transposase genes. Carbapenemase and multiple β-lactam-gene-positive contigs showed high homology (>99% identity) to various plasmids previously described in *K. pneumoniae* (24). Understanding the evolutionary origin of the acquisition of the *K. pneumoniae* resistance gene complement will help track the spread of antibiotic resistance among clinical isolates. Future genome-wide association studies utilizing the catalogued genomic plasticity and antibiotic resistance phenotypes will assist in better defining the pathogenic potential of individual isolates in different models of infection (25, 26).

### Nucleotide sequence accession numbers.

The annotated draft genome sequences for *K. pneumoniae* strains K1, OC217, OC511, OC648, and Z3209 have been deposited in GenBank under accession numbers LOEJ00000000, LOEF00000000, LOEI00000000, LOEH00000000, and LOEG00000000, respectively.
